# In Vitro Contracture Test Results and Anaesthetic Management of a Patient with Emery-Dreifuss Muscular Dystrophy for Cardiac Transplantation

**DOI:** 10.1155/2012/349046

**Published:** 2012-09-03

**Authors:** Frank Schuster, Carsten Wessig, Christoph Schimmer, Stephan Johannsen, Marc Lazarus, Ivan Aleksic, Rainer Leyh, Norbert Roewer

**Affiliations:** ^1^Department of Anaesthesia and Critical Care, University of Wuerzburg, 97080 Wuerzburg, Germany; ^2^Department of Neurology, University of Wuerzburg, 97080 Wuerzburg, Germany; ^3^Department of Cardiothoracic and Thoracic Vascular Surgery, University of Wuerzburg, 97080 Wuerzburg, Germany

## Abstract

Emery-Dreifuss muscular dystrophy (EDMD) is a hereditary neuromuscular disorder characterized by slowly progressive muscle weakness, early contractures, and dilated cardiomyopathy. We reported an uneventful general anaesthesia using total intravenous anaesthesia (TIVA) for cardiac transplantation in a 19-year-old woman suffering from EDMD. In vitro contracture test results of two pectoralis major muscle bundles of the patient suggest that exposition to triggering agents does not induce a pathological sarcoplasmic calcium release in the lamin A/C phenotype. However, due to the lack of evidence in the literature, we would recommend TIVA for patients with EDMD if general anaesthesia is required.

## 1. Introduction

Emery-Dreifuss muscular dystrophy (EDMD), initially considered a benign form of Duchenne or Becker muscular dystrophy, is a hereditary neuromuscular disorder characterized by slowly progressive muscle weakness at the outset with humeroperoneal distribution and early contractures of elbow joints, Achilles tendons, and posterior-cervical muscles. EDMD either presents as an *X-linked disorder* due to a mutation in the emerin gene on chromosome Xq28 or as an *autosomal dominant* form, associated with an aberration of lamin A/C proteins on chromosome 1q11-23 [1]. Emerin and lamin A/C are located in the inner nuclear membrane of different cell types, including skeletal und cardiac muscle fibers. The incidence of autosomal dominant EDMD varies from 1 to 3 : 100.000, while the prevalence of the X-linked recessive form is assumed with 1 in 100.000 males [2]. Cardiac disease occurring by adulthood is a predominant feature of EDMD comprising conduction defects and arrhythmias. Implantation of a pacemaker is recommended if sinus or AV node disease develops [3, 4]. Heart transplantations due to dilated cardiomyopathy and heart failure are rare, but may increase as patients with a pacemaker or cardioverter/defibrillator may have longer survival [5]. 

## 2. Case Report

After obtaining written informed consent of a 19-year-old woman with a lamin A/C associated EDMD scheduled for high urgent cardiac transplantation we reviewed the specific anaesthetic management of this case and presented histological findings of skeletal and heart muscle and results of in vitro contracture-testing.

Beside minor weakness of anterior cervical muscles and proximal upper limbs preoperative neurologic examination was unremarkable. After surviving a sudden cardiac arrest in 2005 an implantable cardioverter defibrillator (ICD) had been inserted. During the last 3 years, she had frequently been hospitalized because of congestive heart failure. Cardiac evaluation revealed an ongoing progression towards dilated cardiomyopathy despite maximum medical therapy. Perioperative echocardiography showed left ventricular and atrial dilatation, an ejection fraction of 22%, moderate tricuspid regurgitation, and an increased systolic pulmonary arterial pressure of 71 mmHg. ECG showed a regular sinus rhythm with incomplete right bundle branch block. After a 3-month waiting period, orthotopic bicaval cardiac transplantation was carried out. Prior to arrival at the operating room, the anaesthetic workstation (Dräger Primus, Germany) was prepared according to our standardized procedure for patients with known malignant hyperthermia susceptibility or muscular disorders: Vapors were removed, carbon dioxide absorbent, fresh gas hose, and breathing circuit were exchanged and the system was flushed with a fresh gas flow of 18 L/min for at least 25 min. Prior to surgery, arterial, central venous, and pulmonary artery catheters for hemodynamic monitoring were inserted under local anaesthesia. Preoperative arterial blood gases (ABG) and metabolic parameters were unremarkable (Table 1). Due to the known muscle dystrophy a total intravenous anaesthesia (TIVA) supplemented with nondepolarising muscle relaxants was carried out. Following 5 min of preoxygenation anaesthesia was induced with 1 *μ*g/kg sufentanil, 2 mg/kg propofol and maintained with continuous propofol (5 mg/kg/h) and sufentanil (1 *μ*g/kg/h) infusion. Rocuronium (0,6 mg/kg) was given to facilitate oral airway intubation and ventilation was adjusted to ABG using low tidal volumes (4–6 mL/kg). Anticoagulation with heparin (500 UI/kg) was monitored by activated clotting time (ACT) in 20 min intervals to maintain ACT > 500 seconds. Tranexamic acid was administered as loading dose of 15 mg/kg within 15 min followed by continuous infusion of 2 mg/kg for 6 hours. During induction of anaesthesia vital signs remained stable. After aortic and bicaval cannulation, cardiopulmonary bypass (CPB) was initiated and cardiac transplantation was performed as described by Shumway and colleagues [6]. Reperfusion phase was prolonged due to reduced donor organ function with an estimated ejection fraction of 30%. Hence, an intra-aortic balloon pump counterpulsation was inserted via the left femoral artery. Afterwards, the patient was weaned from CPB on epinephrine (0.07 *μ*g/kg/min), norepinephrine (0.25 *μ*g/kg/min), enoximone (0.17 mg/kg/h), and inhaled nitric oxide (25 ppm). At this time, a lactic acidosis was noticed, most likely caused by a prolonged episode of low cardiac output (Table 1). Towards sternal closure hemodynamic instability with sudden rise of pulmonal arterial pressure to 60/24 mmHg occurred. The sternum was reopened and inhaled nitric oxide concentration was increased to 34 ppm. Subsequently the patient could be transferred to the ICU in stable condition with an open chest. On the first postoperative day the patient went to the operating room for definite sternal closure and was extubated on the third day after cardiac transplantation. Neurological status after extubation was unaltered to initial findings and the patient was discharged from hospital 30 days after transplantation.

### 2.1. In Vitro Contracture Test and Histological Findings

**Figure 1:**
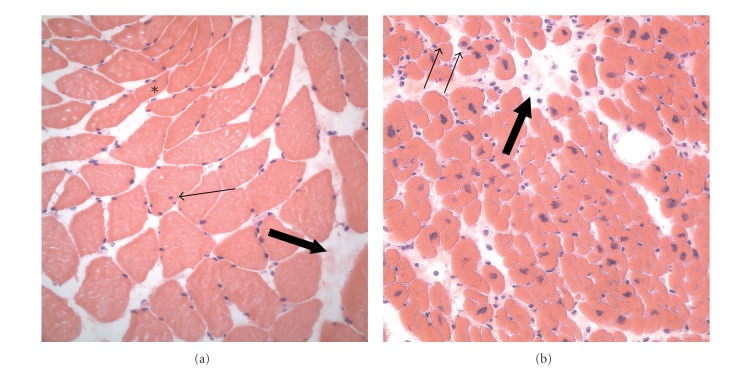
Histology (hematoxylin-eosin staining) of pectoralis major muscle (a) and heart muscle (b). In the pectorals major, mild myopathic changes with hypotrophic fibers (asterisk), some internal nuclei (small arrow), and an increase of fibrous tissue (large arrow) are seen. In the myocardium, there are some atrophic cardiomyocytes (small arrows) and a mild increase in connective tissue.

With prior informed consent of the patient, two bundles of the pectoralis major muscle were excised during the operative procedure. According to the guidelines of the European Malignant Hyperthermia Group an in vitro contracture test was carried out and the muscle bundles were incubated with increasing concentrations of caffeine (0.5; 1; 1.5; 2; 3; 4; 32 mM), respectively, halothane (0.11; 0.22; 0.44; 0.66 mM) [7]. Neither caffeine nor halothane induced significant muscular contractures at the threshold concentrations. There was no evidence of pathologic sarcoplasmic calcium release in response to malignant hyperthermia triggering agents in this patient. Histology of pectoralis major muscle showed mild myopathic changes with slightly increased variation in fiber size, some atrophic fibers, internal nuclei and slight increase of intramuscular fibrous tissue (Figure 1(a)). In the ventricular myocardium many atrophic cardiomyocytes and an increase of connective tissue were detected (Figure 1(b)).

## 3. Discussion

Due to lack of evidence in the current literature, the choice of the best anaesthetic management for patients with muscular dystrophies remains controversial. Several case reports documented the safe application of inhalational agents in patients with Duchenne and Becker type muscular dystrophy [8]. On the other hand, rhabdomyolysis, hyperkalemia and intraoperative or postoperative cardiac arrest occurred in affected patients independently of the use or absence of volatile anesthetics and/or the depolarising muscle relaxant succinylcholine [9]. Explicit recommendations or even guidelines concerning the anaesthetic practice for patients with EDMD are not available. While spinal or epidural anaesthesia were applied without any difficulty for orthopaedic surgery [10–12] and caesarean sections [13, 14], only two cases of uneventful general anaesthesia in EDMD patients using enflurane and succinylcholine [11], or TIVA [15] were described. In contrast to Duchenne and Becker muscular dystrophy, caused by a mutation of the muscle-stabilizing protein dystrophin, EDMD is associated with an aberration of the inner nuclear membrane proteins emerin or lamin A/C. As emerin and lamin A/C are suspected being responsible for the fixation and stabilization of the myonuclei during muscular contraction, alterations of these nuclear membrane proteins may cause defective cellular signalling in response to mechanical stimulations [16]. However, the impact on sarcoplasmic calcium release remains cloudy [17]. The missing muscular response to caffeine and halothane in the performed in vitro contracture test may be a hint that exposition to triggering agents does not lead to a dysfunction of calcium homeostasis in the lamin A/C phenotype. However, we should be cautious transferring in vitro results to in vivo conditions and drawing conclusions based on this single investigation. For safety reasons and in absence of significant publications on anaesthesia in patients with EDMD we decided to use TIVA in our patient. During and after anaesthesia no signs of hypermetabolism or rhabdomyolysis were observed. The lactic acidosis during reperfusion and after weaning from CPB was most likely a result of the initially reduced cardiac output of the transplanted organ, while the increase of serum creatine kinase and myoglobin were comparable to other major surgical procedures [18].

Interestingly, the extent of cardiac involvement does not correlate with the degree of skeletal muscle symptoms. Progressive cardiomyopathy without peripheral muscular symptoms similar to our patient has been previously reported for lamin A/C phenotypes [19]. In affected patients ECG abnormalities, for example, low amplitude P waves, prolonged PQ interval and atrial fibrillation or flutter may occur. These changes may proceed to atrial and ventricular conduction blocks, possibly resulting in complete heart block and necessitating temporary or permanent cardiac pacing. During the course of the disease cardiomyopathy leading to congestive heart failure may develop [4]. In patients with significant cardiomyopathy myocardial depressant agents should be avoided and cardiac pacing must be readily available at any time in the perioperative period.

Early appearance of skeletal muscle contractures before onset of muscular weakness is unique to EDMD. Involvement of posterior-cervical muscles may significantly reduce possible neck flexion and complicate endotracheal intubation, even with a normal Mallampati score [15]. If in doubt awake fiberoptic intubation should be preferred to secure the airway. Furthermore, lumbar paravertebral muscle contractures may hamper the application of spinal or epidural anaesthesia [13].

In summary, we reported an uneventful general anaesthesia using TIVA for cardiac transplantation in a 19-year-old woman suffering from EDMD. The in vitro contracture test results of the patient suggest that exposition to triggering agents does not induce a pathological sarcoplasmic calcium release in the lamin A/C phenotype. However, due to the lack of evidence in the literature, the authors would recommend TIVA for patients with EDMD if general anaesthesia is required.

## Figures and Tables

**Table 1:** Metabolic parameters at different points of time during cardiac transplantation.

	F_*i*_O_2_	pH	PCO_2_ [mmHg]	PO_2_ [mmHg]	Base excess	Laktate [mmol/L]	Creatinkinase [U/L] reference value: <170 U/L	Myoglobin [*μ*g/L] reference value: 25–58 *μ*g/L	Temp. °C
Initial	0.21	7.5	34	62	3.4	0.8	53	30	37.2
Reperfusion	1.0	7.25	44	235	−7.7	8.4	53	56	36.3
After weaning from CPB (central venous)	1.0	7.29	47	59	−7.6	9.5	729	830	36.9
Admission on ICU	0.4	7.32	46	156	−1.9	—	1357	1283	36.3
1 postoperative day	0.35	7.41	48	122	5.8	—	1292	1005	—
